# Cervical Cannulation for Surgical Repair of Congenital Cardiac Defects in Infants and Small Children

**DOI:** 10.21470/1678-9741-2016-0083

**Published:** 2017

**Authors:** Pankaj Garg, Arvind Kumar Bishnoi, Ketav Lakhia, Parth Solanki, Jigar Surti, Komal Shah, Sanjay Patel

**Affiliations:** 1 Department of Cardiovascular and Thoracic Surgery, U. N. Mehta Institute of Cardiology and Research Center (affiliated to BJ Medical College, Ahmedabad), Gujarat, India.; 2 Department of Pediatric Anesthesia, U. N. Mehta Institute of Cardiology and Research Center (affiliated to BJ Medical College, Ahmedabad), Gujarat, India.; 3 Department of Research, U. N. Mehta Institute of Cardiology and Research Center (affiliated to BJ Medical College, Ahmedabad), Gujarat, India.

**Keywords:** Carotid Artery, Common. Jugular Veins. Cardiopulmonary Bypass. Heart Defects, Congenital

## Abstract

**Introduction:**

The biggest challenge faced in minimally invasive pediatric cardiac surgery is cannulation for cardiopulmonary bypass. Our technique and experience of cervical cannulation in infants and small children for repair of congenital cardiac defects is reported in this study.

**Methods:**

From January 2013 to June 2015, 37 children (22 males) with mean age of 17.97±8.63 months and weight of 8.06±1.59 kg were operated on for congenital cardiac defects through right lateral thoracotomy. The most common diagnosis was ventricular septal defect (18 patients). In all patients, right common carotid artery, right internal jugular vein and inferior vena cava were cannulated for institution of cardiopulmonary bypass and aorta was cross clamped through right 2^nd^ intercostal space.

**Results:**

There were no deaths or any major complications related to cervical cannulation. Common carotid artery cannulation provided adequate arterial inflow while internal jugular vein with inferior vena cava provided adequate venous return in all patients. No patient required conversion to sternotomy or developed vascular, neurological or wound related complications. Three patients had residual lesions (small leak across ventricular septal defect patch-2, Grade II left atrio-ventricular valve regurgitation-1) and one patient had mild left ventricular dysfunction. At discharge, both common carotid artery and internal jugular vein were patent on color Doppler ultrasonography in all patients. In a mean follow-up period of 11.4±2.85 months, all patients were doing well. No patient had any wound related, neurological or vascular complication. No patient had residual leak across ventricular septal defect patch.

**Conclusion:**

Cervical cannulation of common carotid artery and internal jugular vein is a safe, reliable, efficient and quick method for institution of cardiopulmonary bypass in minimally invasive pediatric cardiac surgery.

**Table:** 

Abbreviations, acronyms & symbols				
ACT	= Activated clotting time		NIRS	= Near-infrared spectroscopy
ASD	= Atrial septal defect		OS-ASD	= Ostium secundum atrial septal defect
BSA	= Body surface area		PTFE	= Polytetrafluoroethylene
CCA	= Common carotid artery		RA	= Right atrium
CECT	= Contrast enhanced computed tomography		rSO2	= Regional cerebral oxygen saturation
CPB	= Cardiopulmonary bypass		SD	= Standard deviation
ECMO	= Extracorporeal membrane oxygenator		SVC	= Superior vena cava
ICU	= Intensive care unit		TCD	= Transcranial Doppler
IJV	= Internal jugular vein		TEE	= Trans-esophageal echocardiography
IVC	= Inferior vena cava		TTE	= Trans-thoracic echocardiography
LSVC	= Left superior vena cava		VSD	= Ventricular septal defect

## INTRODUCTION

In the last three decades, there has been significant advancement in minimally invasive cardiac surgery in adults^[1]^. In pediatric patients, restricted exposure and complications associated with peripheral vascular cannulation has limited the use of minimally invasive techniques predominantly in larger children and for simple congenital cardiac defects. Whenever performed in infants and smaller children, total central cannulation is the preferred technique^[2,3]^. However, this makes the operative field cluttered and reduces the space for instrument maneuverability.

Over the years, innominate artery and common carotid artery (CCA) cannulation for arterial inflow has been proved as safe technique for commencement of cardiopulmonary bypass (CPB) in pediatric and adult patients, respectively for complex aortic surgeries without complications^[4-6]^. Similarly, direct cannulation of CCA and internal jugular vein (IJV) has been safely used for institution of extracorporeal membrane oxygenator (ECMO) in neonates and infants with minimal risk of long-term vascular complications and stroke. Studies have failed to find any definitive association between CCA ligation and risk of stroke^[7,8]^.

CCA cannulation was performed at our institute in 5 pediatric patients operated for re-do cardiac surgery (unpublished data). CCA cannulation was performed either because patients sustained cardiac injury (three patients) or heart was adherent to sternum on preoperative computed tomography of chest (two patients). In all five patients, femoral and iliac vessels were found unsuitable for cannulation either because of adhesions from previous cannulation or small size of vessels. All five patients had an uneventful recovery without neurological or vascular complication and both CCA and IJV were patent on postoperative Doppler ultrasonography. Using the similar technique of cervical cannulation, infants and small children were operated on with congenital cardiac lesions through a limited right lateral thoracotomy. CPB was instituted by cannulation of CCA, IJV and inferior vena cava (IVC). The aim of this study was to evaluate our technique's clinical usefulness and efficiency in terms of postoperative complications especially neurologic outcomes.

## METHODS

From January 2013 to June 2015, 37 pediatric patients (22 males) with mean age of 17.9±8.63 months and mean weight of 8.06±1.59 kg were operated on for congenital cardiac lesions by lateral thoracotomy using cervical cannulation for CPB. Demographic profile of the patients is shown in Table 1. Forty-three percent of them were infants and 83% weighed <10 kg. The study was approved by the Ethics Committee of our institute. Parents or guardians of the patients were informed in detail about the procedure and written consent was obtained. All patients were operated on by the same surgeon.

**Table 1:** Demographic data of patients.

Variables		N=37
		Mean ± SD
Sex (Male)		22 (60%)
Age (Month)		17.9±8.63 (8-36)
Weight (kg)		8.06 ±1.59 (5.2-13)
**Cardiac Anomaly and Surgical Procedure**		
VSD	Patch Closure	18
OS-ASD	Patch Closure	4
	Direct Closure	7
SV-ASD	Pericardial Patch Repair	4
OP-ASD	Pericardial Patch Closure+ Atrioventricular Valve Repair	4

ASD = atrial septal defect; OP = ostium primum; OS = ostium secundum; SV = sinus venous; VSD = ventricular septal defect.

In all the patients, preoperative cardiac diagnosis was determined by trans-thoracic echocardiography (TTE). Other investigation, like contrast enhanced computed tomography (CECT) and angiography of heart and cardiac catheterization, were performed, if indicated.

### Anaesthetic Management

Central venous catheterization was performed intraoperatively through right femoral vein and arterial access was obtained through right radial artery catheterization. Trans-esophageal echocardiography (TEE) was performed to confirm the diagnosis, de-airing and adequacy for surgical procedure. After induction, bilateral regional cerebral oxygen saturation (rSO_2_) was measured using near-infrared spectroscopy (NIRS) (INVOS 5100B, Somanetics, Inc., Troy, MI) and right sided rSO_2_ was maintained at >90%^[9]^.

### Postoperative Management

All the patients were transferred to intensive care unit (ICU), intubated and were managed as per ICU protocol. Repeat TTE was performed in all the patients before extubation to rule out ventricular dysfunction and pericardial collection and at the time of discharge to assess the ventricular function and rule out any residual lesion. Color Doppler ultrasonography of neck vessels was performed in all the patients in order to assess the patency of CCA and IJV.

### Follow-Up

All the patients were followed up with physical examination in the first week, first month and then in the third month or as required after surgery to assess them clinically for symptoms, wound healing and any restriction of shoulder motility. TTE was performed in all the patients to assess the status of any residual defect and ventricular function.

### Operative Technique

#### Position and thoracotomy

**Fig.1:**
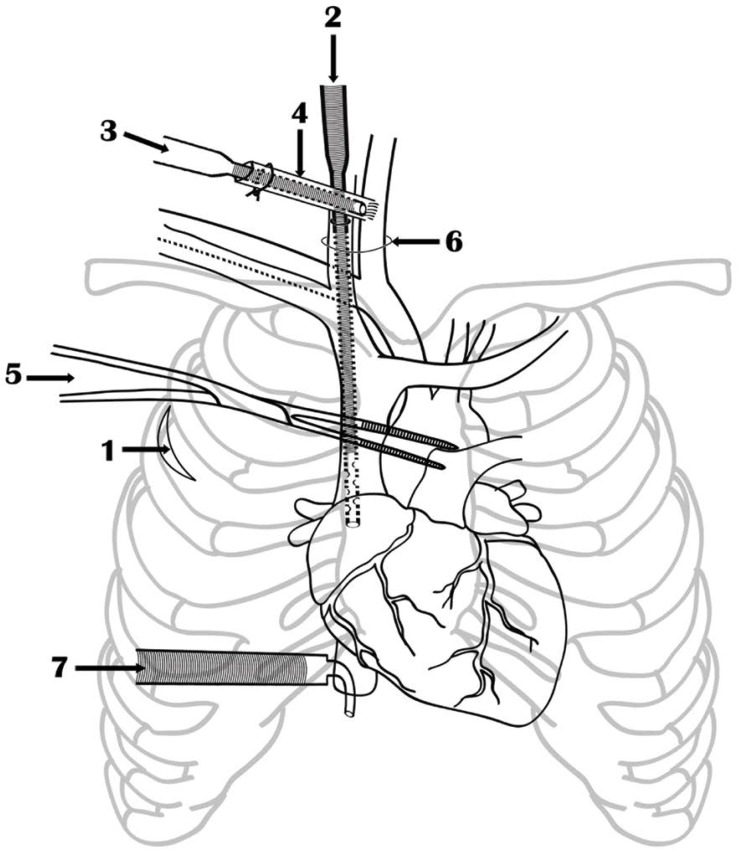
Technique of cervical cannulation of common carotid artery and internal jugular vein, inferior vena cava cannulation and aortic cross clamping using De Bakey coarctation clamp. 1) Lateral thoracotomy incision, 2) Internal jugular vein cannulation, 3) Common carotid artery cannulation, 4) Polytetrafluoroethylene graft, 5) De Bakey coarctation clamp, 6) Cervical skin incision, 7) Inferior vena cava cannula.

Patient lay supine with ring under the head and head turned to the left. Right side of the body was elevated by 30° with a bolster under the right shoulder and right arm was flexed at shoulder and elbow joint to expose the second intercostal space. The patient was draped to expose the base of neck, axilla and chest up to left nipple. A 3-5 cm vertical incision was placed in mid-axillary line in third or fourth intercostal space and after dividing the muscles, pleural space entered. Intercostal space was chosen depending upon the midpoint of the right atrium (RA) on preoperative chest x-ray (Figure 1).

#### Neck Cannulation

**Fig. 2:**
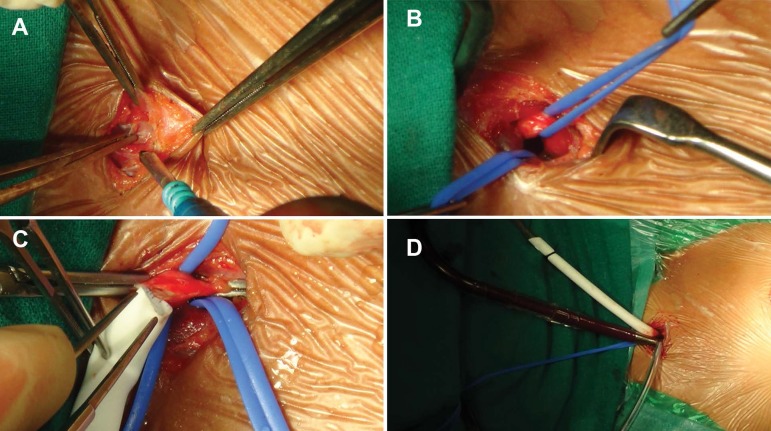
Operative photograph showing sequence of cervical cannulation. (A) Cervical incision (B) Looping of common carotid artery and internal jugular vein (C) Anastomoses of PTFE graft to common carotid artery and (D) Initiation of cardiopulmonary bypass.

A transverse cervical incision 1.5-2 cm in length was made one finger's breadth above the clavicle over the lower part of sternocleidomastoid muscle where two heads of the muscle divide. Subcutaneous tissue and platysma divided and sternocleidomastoid muscle exposed. Mastoid retractor applied and dissection performed between two heads of the muscle. Both, CCA and IJV were isolated in carotid sheath, looped and mobilized along length. Care was taken while mobilizing IJV to avoid induction of spasm, which would make subsequent introduction of venous cannula difficult. Whenever found, a small branch on the medial aspect of IJV was ligated and divided. Rest of the mobilization of both the vessels was safe as they don't have any other branch in the neck. Both the vessels were looped and the patient was heparinized with 400 U/kg. Three minutes later, 5 mm polytetrafluoroethylene (PTFE) graft was anastomosed to CCA artery after clamping (Figure 2) and synthetic cyanoacrylate glue (OMNEX Surgical Sealant, Ethicon Inc, Johnson and Johnson, Somerville, NJ, USA) was applied on the anastomosis to prevent oozing of the blood the needle holes. Aortic cannula was inserted into the graft and tied with silk thread. CCA clamps were removed, arterial cannula de-aired and connected to arterial line. IJV was clamped cranially and opened transversely. Straight venous cannula was inserted and advanced into RA, snugged and connected to venous line. IJV was kept snared cranially during the procedure. Both, arterial and venous cannulas were selected as per body surface area (BSA). In patients with BSA ≤0.5 m^2^, 12Fr DLP femoral arterial cannulae and 12Fr Biomedicus One Piece Femoral Venous Arterial Cannulae (Medtronic Inc. Minneapolis, MN, USA) were used for CCA and IJV cannulation respectively; while in patients with BSA 0.5-0.69 m^2^, similar cannula of 14Fr size were used. For IVC cannulation, DLP single stage venous cannulae with right angle metal tip (Medtronic Inc. Minneapolis, MN, USA) used was one size larger than IJV cannula.

#### Conduct of Cardiopulmonary Bypass

After achieving the adequate activated clotting time (ACT), CPB was initiated and attention was turned to thoracotomy. Mechanical ventilation was disconnected and pericardiotomy was performed parallel and 2-3 cm anterior to phrenic nerve to expose the ascending aorta cranially and IVC caudally and pericardial stays were inserted. Carbon dioxide was continuously insufflated into the operative field throughout the procedure to displace intra-cardiac air. IVC was dissected, looped and cannulated with angled venous cannula that was advanced into the pericardial cavity through a stab incision in subxiphoid area. IVC was snugged and snugger was left in the pleural cavity to keep it out of the operative field. Mild hypothermia (30-32°C) was established. Assisted venous drainage was used, if required, with a median vacuum pressure of 30 mmHg (range, 20-40 mmHg). RA appendage was retracted towards IVC and dissection was performed with scissors between aorta and right pulmonary artery to mobilize the aorta. De Bakey atraumatic coarctation clamp was passed through a stab incision in right second intercostal space in mid-axillary line and aorta was cross clamped at the level of right pulmonary artery. Del Nido cardioplegia was delivered into the aortic root using 16G needle puncture. When cardioplegia delivery was finished, cardioplegia puncture site was enlarged with sharp scissors and a 5-0 plegted polypropylene (prolene) suture was placed around the puncture site and left loose. IJV cannula was pulled back into superior vena cava (SVC). SVC was snugged and snugger was left into the pleural cavity as for IVC snugger. RA was opened and a vent was inserted into the left atrium through patent foramen ovale or atrial septal defect (ASD). When defect was absent, it was created and vent was inserted.

#### Surgical Repair

Repair was performed depending upon the cardiac lesion similar to midline sternotomy approach except in patients with ventricular septal defect (VSD). For repair of VSD, anterior leaflet of the tricuspid valve was detached from annulus to expose the borders of VSD.

In our series, three patients with ostium secundum atrial septal defect (OS-ASD) had associated left superior vena cava (LSVC). In one patient, we were able to loop and snug the LSVC by retracting the aorta and pulmonary artery; it was snugged to prevent the flooding of operative field. In other two patients, straight venous cannula was inserted in the LSVC through coronary sinus and connected to cardiotomy sucker for drainage.

When intra-cardiac repair was finished, RA was repaired and SVC and IVC snuggers were removed. After RA closure, carbon dioxide insufflation was stopped. Ventricular pacing wire was placed. De-airing was performed by gently compressing the heart against the spine with rear end of De Bakey forceps and the lungs were ventilated manually. Aortic root was vented by allowing bleeding from the cardioplegia puncture site into the pericardial cavity. Under low flows, aortic cross clamp was removed and IJV cannula was advanced into RA. Time was allowed for the heart to recover the sinus rhythm with good contractility of the ventricles. After re-warming of the patient, IVC cannula was removed and IVC repaired. After confirming the adequate de-airing on TEE, cardioplegia puncture site was repaired. Finally, CPB was terminated in usual fashion.

When the procedure was finished, IJV cannula was removed and IJV was repaired with interrupted 6-0 prolene sutures. Heparin was reversed with protamine sulfate using dose of 1.0 mg/100 U of heparin. The PTFE graft was severed near the anastomosis with CCA and was oversewn to prevent any residual stump. CCA pulsation was confirmed. After hemostasis, both heads of sternocleidomastoid muscle were approximated. Platysma and subcutaneous tissues were sutured. A pericardial drain inserted through subxiphoid incision and another right pleural drain through axillary incision. Pericardium was loosely approximated. Ribs were loosely approximated with absorbable suture. Subcutaneous tissue and skin were repaired with absorbable sutures.

### Statistical Analysis

Results are expressed as mean ± standard deviation (SD). All data analysis was performed using SPSS for windows, version 20.0 (SPSS Inc., Chicago, IL, USA).

## RESULTS

Thoracotomy was performed in the third intercostal space in 31 patients and in the fourth intercostal space in 6 patients. The lenght of axillary skin incision varied between 3 and 6 cm and of cervical incision between 1.5 and 2 cm. Two patients required enlargement of thoracotomy incision to improve the exposure of VSD.

Intraoperative and postoperative data is shown in Table 2. CCA cannulation provided adequate arterial flow in all patients. Similarly, IJV and IVC cannulation provided adequate venous return except in five patients who required assisted venous drainage. No patient required a switch to sternotomy for arterial or venous return. Furthermore, no intraoperative complications related to the cannulation site, exposure or bleeding was noted. All the patients, except three, regained spontaneous sinus rhythm following removal of aortic cross clamp. Three patients regained ventricular fibrillatory rhythm initially; two spontaneously reverted to sinus rhythm once the heart was completely emptied while one patient required cardioversion.

**Table 2:** Intraoperative and postoperative data including echocardiography and complications.

Variables	N=37 Mean ± SD
Cross clamp time (min)	64.45 ± 29.99 (range)
Bypass time (min)	99.64 ± 30.54 (range)
Assisted venous drainage	5
Inotropic score	4.24 ± 3.15
Mechanical ventilation time (hr)	7.45 ± 1.70 (range)
Drainage output (ml)	13.37 ± 16.01 (range)
ICU stay (day)	1.13 ± 0.34
Hospital stay (day)	3.56 ± 0.55
Follow-up (month)	11.4 ± 2.85
Complications	
Mild MR (Grade II)	1
Mild TR	2
Residual VSD	2
LV dysfunction	1
Wound infection	0
Vascular complications	0
Neurological complications	0

ICU = intensive care unit; LV = left ventricular; MR = mitral regurgitation; TR = tricuspid regurgitation

**Fig. 3:**
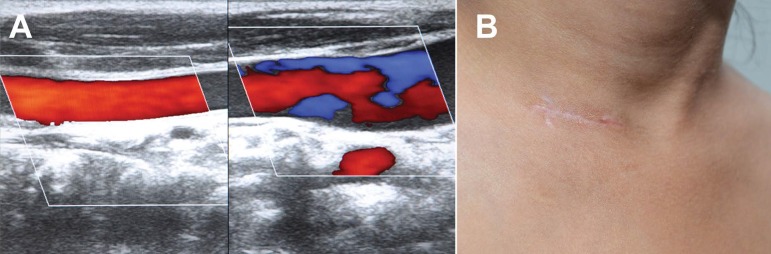
Color Doppler ultrasonography of neck showing patent common carotid artery and internal jugular vein (A) and healed cervical scar (B).

Mean postoperative drain output was 13.37±16.01 ml and no patient required re-exploration for bleeding. Postoperatively, all patients regained consciousness without neurological complication. Mean duration of mechanical ventilation was 7.45±1.7 hours. All patients remained in sinus rhythm and postoperative TTE revealed satisfactory repair without residual defect or ventricular dysfunction. Two patients had 1 mm and 2 mm residual defect across VSD patch, respectively. One patient with ostium primum ASD had grade II left atrioventricular valve regurgitation. Another patient with VSD had mild left ventricular dysfunction. Two patients developed stridor after extubation and were managed conservatively. No patient developed any temporary or permanent, focal or generalized neurological deficit during the hospital stay. Color Doppler ultrasonography of neck vessels confirmed patency of both CCA and IJV in all the patients (Figure 3). There was no operative or hospital mortality. Mean hospital stay was 3.56±0.55 days (3-6 days). All patients were discharged from the hospital in good clinical condition without any cervical or thoracic wound complication.

### Cases where Difficulty was Encountered During Procedure

Three patients who had associated LSVC, difficulty was experienced in accessing the LSVC to cannulate and snug.

### Follow-Up

Follow-up was 100% complete and mean duration of follow-up period was 11.4±2.85 months. During follow-up, all patients were asymptomatic without any evidence of vascular complication or neurologic impairment. All patients had healing of both cervical and thoracotomy wound without keloid formation or shoulder movement deficit. Follow-up TTE confirmed normal left ventricular function in all the patients. In one patient with grade II left atrioventricular valve regurgitation, regurgitation reduced to grade I. None of the patients had tricuspid regurgitation. No residual leak was observed in any of the two patients with leak across VSD patch at the time of discharge.

## DISCUSSION

Direct CCA cannulation has gained popularity for total body circulation as well as antegrade cerebral perfusion during deep hypothermia in adult patients operated on for complex aortic and re-do cardiac surgeries. This technique has proved its efficacy and neurological safety^[4-6]^. In neonates, selective unilateral CCA cannulation is performed by advancing the arterial cannula from ascending aorta or innominate artery to CCA and used for ascending aorta and arch repair during deep hypothermia for antegrade cerebral perfusion^[6]^. Studies have found the technique to be safe without increased risk of neurological complications. As per literatute, direct cannulation of CCA, however, has not been used in pediatric cardiac surgery.

CCA is easily accessible at the base of neck and like innominate artery or aorta, it is sturdy and not easily damaged^[4]^. In our previous experience of 5 patients, CCA was found to be of sufficient size even in small children to supply the total cardiac output. The flow in CCA is distributed to whole body without causing ipsilateral brain hyperperfusion. This is confirmed by the results of our study. We observed adequate radial artery pressures during CPB and absence of any neurological, myocardial or abdominal organ dysfunction in postoperative period in all patients. The possible explanation may lie in an experimental study by Zéicourt et al.^[10]^. Authors using a realistic hypoplastic neonatal aorta template as the base geometry for effect of cannulation technique on flow distribution, found that flow distribution to the different vascular beds in neonates was dictated by the downstream vascular resistances rather than the cannulation strategy. The similar mechanism may be responsible for flow distribution in infants, children and adults as other studies in adults have also failed to find any sign of hyperperfusion with this technique^[5,6]^.

Similarly, IJV is sufficiently large and easily accessible for venous return during CPB. Due to same reasons, cervical cannulation of CCA and IJV are preferred route for establishment of pediatric ECMO. However, in contrast to ECMO, our technique does not require ligation of either CCA or IJV; thus prevents the risk of stroke and cerebral and facial edema^[7]^.

An important concern during CCA cannulation is the risk of neurological injury. Therefore, NIRS was used to detect any decrease in cerebral oxygen saturation from the time of anastomosing the graft to CCA and during CPB. In our study, no patient had >15% fall in NIRS from baseline during CPB. This was probably because the cerebral flow was maintained through both the carotid arteries even during aortic cross clamping as blood supply to whole brain including posterior circulation is maintained by rich network of anastomoses interconnecting the cervical, vertebral, occipital, and carotid arteries designated as 'suboccipital carrefour or knot'^[11]^. Absence of postoperative neurological complications in any patient confirms our assumption.

Cervical cannulation for institution of CPB has several advantages over central cannulation. First, it completely avoids the need to gain control of the aorta for arterial cannulation, an important limitation of lateral thoracotomy^[12]^. As aorta is away from thoracotomy incision, there is poor control of depth of arterial cannula insertion. This increases the risk of vascular accidents as well as neurological complications. Second, cervical cannulation along with aortic cross clamp insertion through a separate stab incision moves the entire hardware away from the operative field. This improves the working space and freedom of instrument maneuverability. Other authors have also emphasized the importance of peripheral cannulation to prevent cluttering in the operative field. However, femoral cannulation is better suited for older children, while cervical cannulation can be performed even in neonates and infants^[12-14]^.

Third, straight multi-hole cannula inserted into IJV is easily advanced into RA and leads to almost complete emptying of the heart. Therefore, mechanical ventilation can be discontinued as soon as CPB is commenced. This improves the exposure of pericardium and heart and ease the insertion and removal of IVC cannula. The IVC cannula can be removed early after removal of aortic cross clamp without compromising the venous drainage. Fourth, this technique of CPB avoids additional groin incisions and potential peripheral vascular complications. Fifth, cannulation of graft rather than direct cannulation of CCA almost nullifies the risk of intimal injury and narrowing of CCA. De-airing can be adequately performed by enlarging the cardioplegia puncture site and TEE guidance. All of our patients readily regained consciousness without neurological complications. This further confirms the adequacy of de-airing of the heart.

An important concern with CCA cannulation is the potential risk of cerebral hyperperfusion during CPB. Transcranial Doppler (TCD) rather than NIRS is better modality to detect the hyperperfusion^[5]^. However, we did not perform the TCD in our study due to non-availability. Our strategy to prevent hyperperfusion was to reduce the arterial pressure during CPB while monitoring the NIRS because cerebral blood flow is predominantly determined by arterial pressure rather than the CPB flow. In our study, no patient developed symptoms of cerebral hyperperfusion, although, we did not specifically investigated this. However, even if brain hyperperfusion is detected on TCD, it can easily be corrected by looping the CCA cranial to graft anastomoses and applying the clip to partially snug the artery to adjust the TCD velocity.

We choose lateral thoracotomy as it is cosmetically and functionally superior^[15]^. However, there are certain shortcoming of this approach *e.g.* difficulty in cannulation of aorta, IVC and limited exposure of the right ventricle free wall and left sided structures of the heart as the heart falls farther away to the left as it gets empty. Cervical cannulation and single dose del-Nido cardioplegia^[16]^ overcome the difficulties associated with cannulation. We, however, believe this incision is suitable only for repair of simple congenital cardiac defects that can be accomplished through RA. In our experience, apical, mid-muscular, doubly committed and outlet VSDs are the contraindications for this approach. We also consider the presence of LSVC as a relative contraindication as it makes the operative procedure cumbersome and the operative field cluttered. We have not operated any patients of tetralogy of Fallot through this approach, however, we consider it a challenge.

Cervical cannulation for CPB can be used with any minimally invasive approach and even in pediatric patients operated for re-do or aortic arch surgeries as in our initial experience. In our experience, this technique adds 15-20 minutes to operative time, however, lots of struggle associated with central cannulation and exposure can be avoided. Further studies with technique may pave the path for this approach as an alternative safe technique for establishment of CPB in pediatric patients.

### Limitations

Limitations of our study include its retrospective nature and small sample size. However, it does contribute to establish cervical cannulation as an alternative technique for CPB in minimally invasive and complex aortic pediatric cardiac surgeries.

## CONCLUSION

Cervical cannulation using side-graft on CCA for arterial inflow and IJV cannulation for venous return is a reliable and safe method for installation of CPB in minimally invasive pediatric cardiac surgery. This technique is safe, efficient, and quick in its execution and compared to minimally invasive technique, this technique avoids the handling of the aorta and the SVC. Complications directly associated with cervical cannulation are uncommon.

**Table:** 

Authors' roles & responsibilities	
PG	Conception and study design; analysis and/or data interpretation; statistical analysis; manuscript redaction or critical review of its content; final manuscript approval
AKB	Conception and study design; analysis and/or data interpretation; statistical analysis; manuscript redaction or critical review of its content; final manuscript approval
KL	Conception and study design; analysis and/or data interpretation; statistical analysis; manuscript redaction or critical review of its content; final manuscript approval
PS	Conception and study design; analysis and/or data interpretation; statistical analysis; manuscript redaction or critical review of its content; final manuscript approval
JS	analysis and/or data interpretation; Conception and study design; statistical analysis; manuscript redaction or critical review of its content; final manuscript approval
KS	Conception and study design; analysis and/or data interpretation; statistical analysis; manuscript redaction or critical review of its content; final manuscript approval
SP	Conception and study design; analysis and/or data interpretation; statistical analysis; manuscript redaction or critical review of its content; final manuscript approval

## References

[1] Iribarne A, Easterwood R, Chan EY, Yang J, Soni L, Russo MJ (2011). The golden age of minimally invasive cardiothoracic surgery:
current and future perspectives. Future Cardiol.

[2] Bauer M, Alexi-Meskishvilli V, Nakic Z, Redzepagic S, Bauer U, Weng Y (2000). The correction of congenital heart defects with less invasive
approaches. Thorac Cardiovasc Surg.

[3] Gundry SR, Shattuck OH, Razzouk AJ, Rio MJ del, Sardari FF, Bailey LL (1998). Facile minimally invasive cardiac surgery via
ministernotomy. Ann Thorac Surg.

[4] Urbanski PP, Lenos A, Lindemann Y, Weigang E, Zacher M, Diegeler A (2006). Carotid artery cannulation in aortic surgery. J Thorac Cardiovasc Surg.

[5] Neri E, Massetti M, Barabesi L, Pula G, Tassi R, Toscano T (2002). Extrathoracic cannulation of the left common carotid artery in
thoracic aorta operations through a left thoracotomy: preliminary experience
in 26 patients. J Thorac Cardiovasc Surg.

[6] Tchervenkov CI, Korkola SJ, Shum-Tim D, Calaritis C, Laliberté E, Reyes TU (2001). Neonatal aortic arch reconstruction avoiding circulatory arrest
and direct arch vessel cannulation. Ann Thorac Surg.

[7] Annich GM, Lynch WR, MacLaren G, Wilson JM, Bartlett RH (2012). ECMO extracorporeal cardiopulmonary support in critical care.

[8] Kurkluoglu M, Hynes CF, Alfares FA, El-Sayed Ahmed MM, Peer SM, Zurakowski D (2015). Choice of peripheral venoarterial extra-corporeal membrane
oxygenation cannulation site in patients above 15 kilograms. J Card Surg.

[9] Zheng F, Sheinberg R, Yee MS, Ono M, Zheng Y, Hogue CW (2013). Cerebral near-infrared spectroscopy monitoring and neurologic
outcomes in adult cardiac surgery patients: a systematic
review. Anesth Analg.

[10] Zéicourt D, Jung P, Horner M, Pekkan K, Kanter KR, Yoganathan AP (2012). Cannulation strategy for aortic arch reconstruction using deep
hypothermic circulatory arrest. Ann Thorac Surg.

[11] Ayad M, Viñuela F, Rubinstein EH (1998). The suboccipital carrefour: cervical and vertebral arterial
anastomosis. AJNR Am J Neuroradiol.

[12] Bacha E, Kalfa D (2014). Minimally invasive paediatric cardiac surgery. Nat Rev Cardiol.

[13] Williams PH, Bhatnagar NK, Wisheart JD (1989). Compartment syndrome in a five-year-old child following femoral
cannulation for cardiopulmonary bypass. Eur J Cardiothorac Surg.

[14] Kadner A, Dave H, Dodge-Khatami A, Bettex D, Vasangiacomo-Buechel E, Turina MI (2004). Inferior partial sternotomy for surgical closure of isolated
ventricular septal defects in children. Heart Surg Forum.

[15] Nguyen K, Chin C, Lee DS, Mittnacht A, Srivastava S, Umesh J (2007). The axillary incision: a cosmetic approach in congenital cardiac
surgery. J Thorac Cardiovasc Surg.

[16] Matte GS, Del Nido PJ (2012). History and use of del Nido cardioplegia solution at Boston
Children's Hospital. J Extra Corpor Technol.

